# Genetic Dissection of Root Morphological Traits Related to Nitrogen Use Efficiency in *Brassica napus* L. under Two Contrasting Nitrogen Conditions

**DOI:** 10.3389/fpls.2017.01709

**Published:** 2017-09-29

**Authors:** Jie Wang, Xiaoling Dun, Jiaqin Shi, Xinfa Wang, Guihua Liu, Hanzhong Wang

**Affiliations:** Key Laboratory of Biology and Genetic Improvement of Oil Crops, Ministry of Agriculture, Oil Crops Research Institute of the Chinese Academy of Agricultural Sciences, Wuhan, China

**Keywords:** root morphology, nitrogen use efficiency, genetic relationship, QTL clusters, rapeseed

## Abstract

As the major determinant for nutrient uptake, root system architecture (RSA) has a massive impact on nitrogen use efficiency (NUE). However, little is known the molecular control of RSA as related to NUE in rapeseed. Here, a rapeseed recombinant inbred line population (BnaZNRIL) was used to investigate root morphology (RM, an important component for RSA) and NUE-related traits under high-nitrogen (HN) and low-nitrogen (LN) conditions by hydroponics. Data analysis suggested that RM-related traits, particularly root size had significantly phenotypic correlations with plant dry biomass and N uptake irrespective of N levels, but no or little correlation with N utilization efficiency (NUtE), providing the potential to identify QTLs with pleiotropy or specificity for RM- and NUE-related traits. A total of 129 QTLs (including 23 stable QTLs, which were repeatedly detected at least two environments or different N levels) were identified and 83 of them were integrated into 22 pleiotropic QTL clusters. Five RM-NUE, ten RM-specific and three NUE-specific QTL clusters with same directions of additive-effect implied two NUE-improving approaches (RM-based and N utilization-based directly) and provided valuable genomic regions for NUE improvement in rapeseed. Importantly, all of four major QTLs and most of stable QTLs (20 out of 23) detected here were related to RM traits under HN and/or LN levels, suggested that regulating RM to improve NUE would be more feasible than regulating N efficiency directly. These results provided the promising genomic regions for marker-assisted selection on RM-based NUE improvement in rapeseed.

## Introduction

As the key element of protein, nucleic acid etc., nitrogen (N) is one of the essential mineral nutrients for crop growth and development. Application of sufficient synthetic N fertilizer at the appropriate time can significantly improve crop yield and quality, but only 30–50% of the applied N fertilizer is taken up from soil by crops (Smil, 1999; Cassman et al., 2002). The overuse of N fertilizer globally causes an increased cost as well as serious environmental problems, such as soil acidification, groundwater and air pollution, and so on (Zhu and Chen, 2002; Good et al., 2004; Galloway et al., 2008; Wuebbles, 2009; Guo et al., 2010). Improving the N use efficiency (NUE) and reducing residual N in environment is therefore the longstanding, worldwide challenge for economical and sustainable agricultural production.

In general, NUE referring to plant productivity can be divided into two components: N uptake efficiency and N utilization efficiency (Good et al., 2004; Chardon et al., 2010). Root system is the key place for nutrient and water acquisition and its ability on soil exploring is the major determinant of N uptake efficiency (Li X. et al., 2016). Root system architecture (RSA), often defined as the spatial configuration and distribution of root system in the growth medium, determines the soil exploration in time and space (Lynch, 1995). Considerable researches have shown that RSA was closely related with N uptake, for example, plants with a steeper and deeper root can absorbs N more efficiently in deep soil layers (Lynch, 2013; Trachsel et al., 2013; Zhan and Lynch, 2015). As an important part of RSA, root morphology (RM, including root length, root surface area, root number etc.) plays an important role in crop N acquisition under field or artificial growth medium condition (Liu et al., 2008; Li et al., 2015; Mu et al., 2015). Correspondingly, RM is in turn significantly influenced by N availability. Therefore, heterogeneity of N supply permits plants to optimize N acquisition from growth medium by modulating their root morphology (Linkohr et al., 2002; Walch-Liu et al., 2006; Yu et al., 2014; Li P. et al., 2016).

Both NUE and root system are complex traits and cannot be measured directly; especially the hidden feature of root makes it difficult to be investigated. Thus, assessment the features of NUE and root system under field conditions for conventional genetic improvement is slow, imprecise and expensive (Hochholdinger and Tuberosa, 2009). In recent decades, various glasshouse- or laboratory-based new technologies (such as hydroponic culture, agar plate culture and paper culture system) have been developed and applied for seedlings root system studying (Yang et al., 2010; Shi et al., 2013; Li P. et al., 2016). Furthermore, quantitative trait locus (QTL) analysis based on genome-wide molecular linkage mapping has become a powerful approach for dissecting the genetic basis of root system as related to nutrient-deficiency tolerance, including several RM-related traits such as lateral root density, total/mean lateral root length, crown/seminal root number, etc. (Zhang et al., 2014, 2016; Li et al., 2015; Gu et al., 2016). With the easiness of phenotype identification, those glasshouse- or laboratory-based new technologies were successfully applied for QTL mapping of root traits and/or NUE traits (Coque et al., 2008; Li et al., 2015). For example, many studies on QTL analysis for RM and/or NUE under low-nitrogen (LN) and high-nitrogen (HN) conditions have been reported in rice (Huang et al., 2004; Lian et al., 2005), maize (Coque et al., 2008; Liu et al., 2008; Li et al., 2015; Li P. et al., 2016; Pestsova et al., 2016), wheat (An et al., 2006), barley (Hoffmann et al., 2012), and cotton (Shang et al., 2016). Hydroponic culture system was used for most of the above studies, while agar plate culture system (Huang et al., 2004) and paper culture system (Pestsova et al., 2016) were applied for the rest two studies. Partial of these studies uncovered a significantly genetic relationship between RM and NUE traits and provided the most promising genomic regions for marker-assisted selection of RM to improve NUE. Importantly, Li et al. (2015) identified 53 advanced backcross-derived lines (ABLs) containing RSA-NUE QTL clusters (root traits included seminal root length, crown/seminal root number, etc.) via marker-assisted selection and the grain yield (GY)/NUE of these ABLs showed apparent mean increases of 13.8% under HN and of 15.9% under LN conditions, compared to recurrent background Wu312, providing a successful study case for the manipulation of RM to improve NUE via marker-assisted selection QTLs in maize.

Rapeseed (*Brassica napus* L., AACC, 2n = 38) is one of the most important oil crops in the world, whereas N fertilization is a limiting factor in rapeseed productivity (Rathke et al., 2005). Understanding the genetic control of RM and improving N uptake ability in low N environment is believed to be an effective way to improve NUE and maintain sustainable production in rapeseed. Although, several root traits QTLs as related to phosphorus uptake efficiency (Yang et al., 2010; Shi et al., 2013; Zhang et al., 2016) and QTLs of NUE traits at agronomic and physiological levels (Bouchet et al., 2014, 2016) have recently been identified, few QTLs of root morphological traits responsible for NUE have been reported in rapeseed. These hinder the progress in nitrogen-efficient genetic improvement through marker-assisted selection of effective RM in rapeseed. In the present study, a rapeseed recombinant inbred line (RIL) population (BnaZNRIL) was used to investigate RM- and NUE-related traits of seedling plants in hydroponics under two contrasting N levels. The main objectives of this study were as follows: (i) to investigate the genetic relationship between RM and NUE under HN and LN conditions; (ii) to identify QTL clusters with pleiotropy or specificity for RM- and NUE-related traits through QTL mapping and QTL meta-analysis; (iii) to provide the promising genomic regions for marker-assisted selection on NUE improvement in rapeseed. Further, our study proposed two distinct NUE-improving approaches (RM-based and N utilization-based directly) in rapeseed, and indicated that RM-based NUE improvement would be more effective than NUE improvement directly via marker-assisted selection in rapeseed.

## Materials and methods

### Plant materials

The BnaZNRIL population, consisting of 184 F7 lines, was derived by single-seed descent from a cross between two sequenced rapeseed cultivars, Zhongshuang11 (*de novo* sequencing) and No. 73290 (resequencing) (Yang et al., 2016). As 9 lines were removed from this population for the lack of seeds, 175 lines plus the two parents were used in this study.

### Hydroponics experiments

Plump and uniform rapeseed seeds were sowed on medical gauze that was fixed to a blue plastic basin (60 × 40 × 15 cm, length × width × height) filled with quarter-strength modified Hoagland's solution (Hoagland and Arnon, 1950). The modified Hoagland's solution (the concentration of N was 15 mM) consisted of: 5 mM Ca(NO_3_)_2_·4H_2_O, 5 mM KNO_3_, 2 mM MgSO_4_·7H_2_O, 1 mM KH_2_PO_4_, 0.05 mM EDTA-Fe, 46 μM H_3_BO_3_, 9.14 μM MnCl_2_·4H_2_O, 0.77 μM ZnSO_4_·7H_2_O, 0.37 μM NaMoO_4_·2H_2_O, and 0.32 μM CuSO_4_·5H_2_O. Six days after sowing as described by Dun et al. (2016), uniform seedlings were selected and transplanted into smaller blue plastic basins (34 × 26 × 12 cm, length × width × height) containing quarter-strength nutrient solution (two N treatments, HN and LN) under the natural condition with a removable rain-shelter. Each basin contained 24 seedlings of 4 lines (six seedlings for each line). As a total, 90 basins were used in each independent experiment. Nutrient solution was renewed once a week. For HN treatment, the quarter-strength and half-strength nutrient solution was used at the first 2 weeks respectively, and full-strength nutrient solution was used until harvest. For LN treatment, the concentration of N was adjusted to 0.5 mM by reducing KNO_3_ and replacing Ca(NO_3_)_2_ by CaCl_2_, while K^+^ was complemented by adding K_2_SO_4_ (refer to Stahl, 2015). The pH value was adjusted to 5.8 ± 0.2 with NaOH or HCl.

Three independent hydroponic culture experiments with a completely random design were carried out at Oil Crops Research Institute of the Chinese Academy of Agricultural Sciences, Wuhan, PR China. The plants were harvested with five fully expanding leaves under HN condition (~37 days after sowing). Accordingly, the plants under LN condition showed typical N-deficiency symptoms. The first experiment (E1) was carried out during the period from October 18 to November 23 in 2015 (37 days), the second (E2) from March 7 to April 14 in 2016 (39 days), and the third (E3) from October 2 to November 4 in 2016 (34 days). During the three experiments, the average temperatures and the average humidities in Wuhan were 19/11°C, 19/10°C, 21/15°C (day/night), and 79, 78, 83%, respectively. Besides, the total sunshine times in Wuhan were 158, 187, and 207 h, respectively, among the three experiments and the average light intensities were about 600–800 μmol•m^−2^•s^−1^.

### Phenotypic investigation

At harvest, four uniform plants of each line were removed from the basin. After they had been sampled, the total roots were separated from the shoot base and primary root length (PRL) was investigated manually using a ruler, while shoots were over dried at 80°C until a constant weight to evaluate shoot dry weight (SDW). The intact root system were immersed and dispersed in a transparent plastic tray with water for scanning with a scanner (EPSON V700, Japan), and total root length (TRL), total root surface area (TSA), total root volume (TRV), total root number (TRN) were analyzed using WinRHIZO software (Pro, 2012b, Canada). Finally, roots were over dried at 80°C to evaluate root dry weight (RDW). Besides, total dry weight (TDW) and root-shoot ratio in dry weight (RSRD) were calculated.

The dried root and shoot samples were separately ground into powder, about 0.1 g were weighted and dissolved in H_2_SO_4_-H_2_O_2_, and then diluted with pure water to 1.25 L. Subsequently, N concentration (mg/L) were analyzed using Smartchem 200 automatic analyzer (Westco Scientific Instruments, Westco). Each sample was measured with three repetitions. RNC or SNC was N content (mg) in per unit weight (g) of root or shoot sample respectively and was calculated as follows: RNC or SNC = (sample N concentration × 1.25) / sample weight. The N uptake of root (RNU) and shoot (SNU) were calculated by multiplying weight by N concentration, respectively. Total N uptake (TNU) or N utilization efficiency (NUtE) was obtained by adding RNU and SNU together or by dividing TDW by TNU. All the traits investigated in this study were summarized in Table 1.

**Table 1:** Summary of the investigated 15 traits in this study.

**Classification**	**Trait**	**Abbreviations**	**Units**	**Trait measurements**
RM-related traits	Primary root length	PRL	cm	Measured with a ruler
	Total root length	TRL	cm	Analyzed by WinRHIZO
	Total root surface area	TSA	cm^2^	Analyzed by WinRHIZO
	Total root volume	TRV	cm^3^	Analyzed by WinRHIZO
	Total root number	TRN	number	Analyzed by WinRHIZO
	Root dry weight	RDW	g	Dried and weighted using a balance (1/1,000 g)
	Root-shoot ratio in dry weight	RSRD	g/g	RDW/SDW
NUE-related traits	Shoot dry weight	SDW	g	Dried and weighted using a balance (1/100 g)
	Total dry weight	TDW	g	RDW + SDW
	Shoot N concentration	SNC	mg/g	Smartchem 200 automatic analyzer
	Root N concentration	RNC	mg/g	Smartchem 200 automatic analyzer
	Shoot N uptake	SNU	mg	SNC × SDW
	Root N uptake	RNU	mg	RNC × RDW
	Total N uptake	TNU	mg	RNU + SNC
	N utilization efficiency	NUtE	g/mg	TDW/TNU

### Data analysis

A total of 15 phenotypic traits, including 7 RM- and 8 NUE-related traits (Table 1) represented by the means of four plants for each genotype, were used for phenotypic analysis and QTL analysis. The broad-sense heritability (h^2^) of each measured trait was calculated as h^2^ = $\sigma_{\text{g}}^{2}$/($\sigma_{\text{g}}^{2}$ + $\sigma_{\text{ge}}^{2}$/n + $\sigma_{\text{e}}^{2}$/nr), where $\sigma_{\text{g}}^{2}$, $\sigma_{\text{ge}}^{2}$, and $\sigma_{\text{e}}^{2}$ are the variance of genotype, genotype × environment, and error, respectively, and n and r are the number of independent experiments and replications, respectively. The estimation of $\sigma_{\text{g}}^{2}$, $\sigma_{\text{ge}}^{2}$, and $\sigma_{\text{e}}^{2}$ were obtained with the software SAS 9.2 (SAS Institute Inc., NC, USA) using the GLM procedure. Correlation analysis and principal component analysis (PCA) were calculated with software SAS 9.2 (SAS Institute Inc., NC, USA) using the PROC CORR and PROC PRINCOMP procedure, respectively.

The linkage mapping of QTLs was performed by composite interval mapping program (Zeng, 1994) using the Windows QTL Cartographer version 2.5 software (http://statgen.ncsu.edu/qtlcart/WQTLCart.htm). The corresponding genetic map consisted of 2264 unique loci/bins, which covered a total length of 2,107 cM distributed on 19 linkage groups (Yang et al., 2016). In this study, a walk speed of 1 cM, 5 control markers, a window size of 10 cM and forward regression method were used. The LOD threshold was determined by permutation analysis with 1,000 repetitions (Churchill and Doerge, 1994). The LOD threshold was set at 3.2–6.2 (*p* = 0.05) to identify significant QTLs. To avoid missing QTLs with a relatively small effect, a lower LOD threshold was set at 1.7–2.2 (*p* = 0.50) to detect suggestive QTLs. Both significant QTLs and overlapping suggestive QTLs were admitted (Long et al., 2007) and named as “identified QTL” (Shi et al., 2009).

In this study, “stable QTL (sQTL)” was defined, which was repeatedly detected with more than half of 2-LOD confidence interval overlapping at least two environments or different N levels and has the same additive-effect direction. In addition, sQTLs detected under both HN and LN conditions were called as “constitutive sQTL,” while sQTLs that were detected in at least two environments under either HN or LN conditions, respectively were named as “HN-specific sQTL” or “LN-specific sQTLs,” respectively (Li et al., 2015). These sQTLs were divided into two types: QTLs detected at least once with phenotypic variation explained (R^2^) ≥ 20% or at least twice with R^2^ ≥ 10% were named as “major sQTL”, and the remainder were named as “minor sQTL” (Price, 2006; Maccaferri et al., 2008; Shi et al., 2009). And QTL meta-analysis was performed using BioMercator 4.2 software (Arcade et al., 2004) to estimate the coincidences of several QTLs for different traits (at least two different traits), which were integrated into the “QTL cluster.” For a QTL cluster, the coincidence of QTLs for two or more traits with same additive-effect directions was considered to be positive, while the coincidence of QTLs with opposite additive-effect directions was considered negative (Coque et al., 2008).

Each identified QTL or QTL cluster was named as “q” + “the name of the trait abbreviation” or “qc” + “the linkage group,” respectively. Arabic numerals were added, if more than one QTL cluster was located on the same linkage group.

## Results

### Phenotypic variation for RM- and NUE-related traits in the BnaZNRIL mapping population of “Zhongshuang11” × “No. 73290” under two contrasting N levels

Three independent hydroponic experiments for the parental lines and the RIL population were performed to evaluate 7 RM- and 8 NUE-related traits (Table 1) under both HN and LN growth conditions. Although the two parents (Zhongshuang11 and No.73290) showed no significant difference for most of RM- and NUE-related traits under HN level (except in E2, Zhongshuang11 showed significant higher values than No. 73290 in RDW, TRL, TSA, TRV, RNU, SNU, and TNU), they displayed significant differences for PRL, TRL, TSA, TRV, TRN, RDW, and NUtE in all three environments or a single environment under LN level (Table S1).

Continuous phenotypic distribution values with obvious kurtosis among these lines suggesting a quantitative inheritance pattern suitable for QTL identification. Minimum, maximum, mean values and coefficient of variations (CVs) for all investigated traits from each experiment were listed in Table 2. Most traits had considerable phenotypic variation within the BnaZNRIL population as suggested by the CVs ranging from 7.6 to 33.8% (Table 2). Increased PRL (1.6–89.9%), RSRD (54.5–172.7%), TRL (5.2–14.8%), and NUtE (11.5–200%) were response to LN stress, compared with those under HN levels (Table 2), indicating that LN stress stimulated root growth for more N nutrition uptake and improved N utilization, though shoot growth was obviously inhibited. The heritability (h^2^) of RM-related traits was relatively high, ranging from 0.38 to 0.67 under HN and from 0.48 to 0.60 under LN condition (Table 2). However, the heritability (h^2^) of NUE-related traits was moderate except SNC and NUtE (low under HN condition, 0.07 and 0.06, respectively), ranging from 0.23 to 0.48 under HN and from 0.26 to 0.59 under LN condition (Table 2). This indicated that RM-related traits are less affected than NUE-related traits on changeable environment under both HN and LN conditions. Although significant genotype × environment was observed, the relatively high heritability (h^2^) in the majority of the investigated traits indicated the genetic stability of these traits in a genotype among the three repetitions.

**Table 2:** Statistics for all investigated traits of BnZNRIL lines across three independent experiments (E1-E3).

**Category**	**Trait**	**Treatment**	**E1**				**E2**				**E3**				**h^2^(%)^b^**
			**Range**	**Mean**	**CV (%)**	**% of Reduction^a^**	**Range**	**Mean**	**CV (%)**	**% of Reduction**	**Range**	**Mean**	**CV (%)**	**% of Reduction**	
RM-related traits	PRL	HN	12.3–31.6	19.4	16.5	22.2	9.1–21.8	15.9	15.2	89.9	10.8–29	18.9	17.1	1.6	0.67
		LN	17–32.3	23.7	14.2		20.4–39.7	30.2	12.1		10.7–28.4	19.2	16.6		0.55
	TRL	HN	696.7–1938.3	1,358.4	17.8	7.2	914.5–3,268.8	2,006.3	21.9	14.8	575.3–1,522.9	929.7	19.2	5.2	0.53
		LN	695.4–2258	1,456.2	18.7		1,372.7–3,543.5	2,302.7	16.6		541.2–1,633.8	978.4	23.3		0.6
	TSA	HN	75.8–165.3	116.4	15.1	0.6	78.5–289.9	182	22.1	−15.2	46.6–118.9	68.8	17.7	1.5	0.48
		LN	66.9–177.4	117.1	15.9		99.4–235.2	154.4	15.1		40–113.8	69.8	22.8		0.58
	TRV	HN	0.49–1.15	0.8	14.6	−6.3	0.54–2.16	1.32	23.8	−37.1	0.24–0.76	0.41	22.2	−2.4	0.45
		LN	0.49–1.14	0.75	15.4		0.52–1.24	0.83	15.6		0.23–0.66	0.4	24.3		0.53
	TRN	HN	467.3–1129.7	797.9	18.9	−6.0	670.3–3,410.3	1,727.5	30.6	−12.5	380–1,094	581.1	19.5	10.3	0.43
		LN	452.3–1342	749.7	19.4		941.8–2,367.8	1,510.9	17		353.3–1,075	641.2	18.1		0.56
	RDW	HN	0.022–0.055	0.038	18	0.0	0.031–0.17	0.098	26.6	−36.7	0.017–0.063	0.029	25.8	−3.4	0.38
		LN	0.024–0.053	0.038	15.7		0.044–0.099	0.062	15.3		0.016–0.048	0.028	23.9		0.57
	RSRD	HN	0.06–0.17	0.09	14.1	88.9	0.06–0.19	0.11	17.9	172.7	0.07–0.17	0.11	16.3	54.5	0.52
		LN	0.11–0.25	0.17	13.1		0.2–0.48	0.3	15.6		0.12–0.23	0.17	13.2		0.48
NUE-related traits	SDW	HN	0.18–0.64	0.43	17.5	−46.5	0.23–1.56	0.92	24.6	−77.2	0.13–0.49	0.26	27.7	−34.6	0.42
		LN	0.12–0.32	0.23	15		0.14–0.33	0.21	15.1		0.08–0.3	0.17	25.5		0.58
	TDW	HN	0.206–0.684	0.468	17.2	−42.3	0.264–1.688	1.018	24.3	−73.0	0.144–0.543	0.294	27.1	−33.7	0.41
		LN	0.154–0.373	0.27	14.6		0.185–0.431	0.275	13.7		0.1–0.34	0.195	24.9		0.59
	SNC	HN	25.66–49.73	39.47	8.6	−7.7	24.46–47.45	36.86	9.6	−67.7	20.18–53.55	43.46	7.7	−14.1	0.07
		LN	27.96–48.75	36.44	9.6		8.04–16.55	11.92	14		24–49.66	37.33	13.8		0.40
	RNC	HN	23.42–57.95	32.3	12.8	−19.0	21.36–61.25	37.86	17.4	−62.4	32.56–70.26	40.27	11.2	−11.9	0.23
		LN	18.37–30.97	26.17	7.6		8.27–19.48	14.23	14.2		20.18–42.73	35.47	10.4		0.34
	SNU	HN	6.86–26.8	16.98	19.8	−50.4	8.5–58.93	34.04	27.6	−92.6	5.12–26.42	11.45	28.6	−46.1	0.32
		LN	4.94–11.15	8.42	14.5		1.51–4.54	2.53	20.2		3.29–11.68	6.17	20.8		0.3
	RNU	HN	0.59–1.85	1.23	21.6	−19.5	1.09–7.89	3.74	33.8	−76.5	0.69–2.66	1.17	27.8	−15.4	0.48
		LN	0.65–1.37	0.99	16.4		0.43–1.59	0.88	19.3		0.6–1.62	0.99	21		0.43
	TNU	HN	7.64–28.59	18.19	19.6	−48.3	9.6–62.47	37.87	26.6	−91.0	5.83–28.51	12.64	27.9	−43.1	0.31
		LN	5.74–12.24	9.4	13.9		2.08–5.71	3.41	16.9		3.89–13.06	7.19	19.4		0.32
	NUtE	HN	0.021–0.038	0.026	8.9	11.5	0.021–0.037	0.027	9.5	200.0	0.019–0.046	0.023	9.6	17.4	0.06
		LN	0.023–0.036	0.029	8.7		0.06–0.111	0.081	11.6		0.021–0.04	0.027	13.3		0.26

a *% of Reduction on Mean = (LN-HN)/HN × 100*.

b *Broad-sense heritability*.

Further, for each trait, part of RIL lines had values outside the range between parental lines, suggesting the presence of alleles with positive effects for root development and N utilization in both parents. For individual RM- or NUE-related traits, several of the transgressive lines always displayed extreme values lower or higher than parental lines over the three experiments (for example, B031 had 7–32% lower TRL and RDW than No. 73290, while B152 had 11–41% higher TRL and RDW than ZS11 under LN conditions), providing useful lines for RM or NUE genetic improvement and corresponding molecular mechanism dissection.

### Genetic relevance between RM- and NUE-related traits in the BnaZNRIL mapping population

The phenotypic correlations among all examined traits were calculated and the principal component analyses (PCA) for all investigated traits were performed to reveal the genetic correlation among RM- and NUE-related traits. For RM-related traits, the majority of traits had strong and significantly correlations (*r* = 0.71–0.93 under HN levels and *r* = 0.61–0.95 under LN conditions, *P* < 0.0001) with each other irrespective of N levels with the exception of PRL and RSRD (Table 3), indicating developmental relevance among these RM-related traits. PRL and RSRD showed smaller or no significant correlation with other RM-related traits under HN condition (*r* = 0.01–0.44 for PRL, *r* = 0.02–0.13 for RSRD with others). However, PRL and RSRD had significantly correlations with other RM-related traits under LN condition (*r* = 0.17–0.54 for PRL, *P* < 0.05; *r* = 0.25–0.38 for RSRD, *P* < 0.05; except r between RSRD and TRN) (Table 3), revealing the change of root system response to N stress, including PRL increasing and faster root development. For NUE-related traits, the dry biomass traits (DB, including SDW and TDW) had high values of correlation coefficients with N uptake traits (NU), SNU (*r* = 0.82–0.95), RNU (*r* = 0.61–0.71), and TNU (*r* = 0.77–0.95) irrespective of N levels, as expected (Table 3), suggesting that plant growth depend heavily on N uptake. On other hand, DB had no significant correlation with NUtE under HN level, while positive correlation with NUtE (*r* = 0.28) under LN level, indicating that plants rely on the higher N uptake and NUtE to maintain larger biomass under LN level (Table 3).

**Table 3:** Phenotypic correlation coefficients (r) among all investigated traits in the BnaZNRIL population grown under HN and LN levels.

**Treatment**	**Category**	**Traits**	**RM-related traits**							**NUE-related traits**						
			**PRL**	**TRL**	**TSA**	**TRV**	**TRN**	**RDW**	**RSRD**	**SDW**	**TDW**	**SNC**	**RNC**	**SNU**	**RNU**	**TNU**
HN	RM-related traits	TRL	0.44													
		TSA	0.24	0.93												
		TRV	0.01	0.73	0.93											
		TRN	0.17	0.84	0.87	0.78										
		RDW	0.04	0.71	0.87	0.90	0.80									
		RSRD	−0.02	0.02	0.08	0.11	0.10	0.13								
	NUE-related traits	SDW	0.07	0.62	0.71	0.71	0.61	0.75	−0.49							
		TDW	0.07	0.64	0.74	0.74	0.65	0.79	−0.44	1.00						
		SNC	0.09	0.07	0.01	−0.05	0.07	−0.15	−0.06	−0.05	−0.06					
		RNC	0.04	0.02	0.09	0.14	0.06	0.19	−0.04	0.17	0.18	−0.11				
		SNU	0.08	0.61	0.69	0.67	0.61	0.68	−0.48	0.95	0.94	0.21	0.14			
		RNU	0.03	0.51	0.67	0.73	0.62	0.86	0.13	0.61	0.65	−0.22	0.57	0.53		
		TNU	0.08	0.61	0.70	0.69	0.63	0.73	−0.43	0.95	0.95	0.15	0.19	0.98	0.63	
		NUtE	−0.11	−0.11	−0.05	0.01	−0.11	0.08	0.11	−0.04	−0.03	−0.94	−0.10	−0.29	0.08	−0.22
LN	RM-related traits	TRL	0.52													
		TSA	0.38	0.95												
		TRV	0.17	0.79	0.94											
		TRN	0.54	0.79	0.75	0.61										
		RDW	0.25	0.79	0.86	0.85	0.66									
		RSRD	0.00	0.25	0.29	0.29	0.12	0.38								
	NUE-related traits	SDW	0.23	0.58	0.63	0.61	0.55	0.66	−0.41							
		TDW	0.24	0.65	0.70	0.68	0.60	0.75	−0.29	0.99						
		SNC	−0.10	−0.30	−0.26	−0.20	−0.19	−0.29	0.09	−0.43	−0.42					
		RNC	−0.04	−0.26	−0.23	−0.17	−0.13	−0.25	−0.22	−0.10	−0.14	0.34				
		SNU	0.10	0.43	0.53	0.57	0.44	0.53	−0.34	0.83	0.82	0.03	0.08			
		RNU	0.12	0.62	0.72	0.77	0.52	0.82	0.21	0.64	0.71	−0.22	0.20	0.60		
		TNU	0.10	0.45	0.54	0.59	0.46	0.55	−0.26	0.77	0.77	0.03	0.12	0.93	0.66	
		NUtE	0.16	0.27	0.21	0.12	0.16	0.23	−0.01	0.28	0.28	−0.69	−0.48	−0.07	0.04	−0.19
Colored scales:		Negative correlation (*p* < 0.01)				Negative correlation (*p* < 0.05)				Positive correlation (*p* < 0.05)			Positive correlation (*p* < 0.01)			

*Upper: Pearson correlation coefficients (r) under HN levels; Lower: Pearson correlation coefficients (r) under LN levels. The significant correlation is highlighted with colors: positive correlation is indicated in red, and negative correlation in blue. Colored scales represented different levels of coefficients*.

Pearson's correlations were also calculated comparing the RM- and NUE-related traits. It was obviously that RM-related traits (except for PRL and RSRD) was significant correlated with NU and DB irrespective of N levels (*r* = 0.51–0.86 under HN condition, *r* = 0.43–0.82 under LN condition) (Table 3), indicating the phenotypic relationship between root system, plant growth and N use efficiency. Interestingly, although SNC and RNC showed no significant correlation with RM-related traits under HN condition, they are significantly correlated with several RM-related traits (TRL, TSA, and TRV) under LN condition.

**Figure 1:**
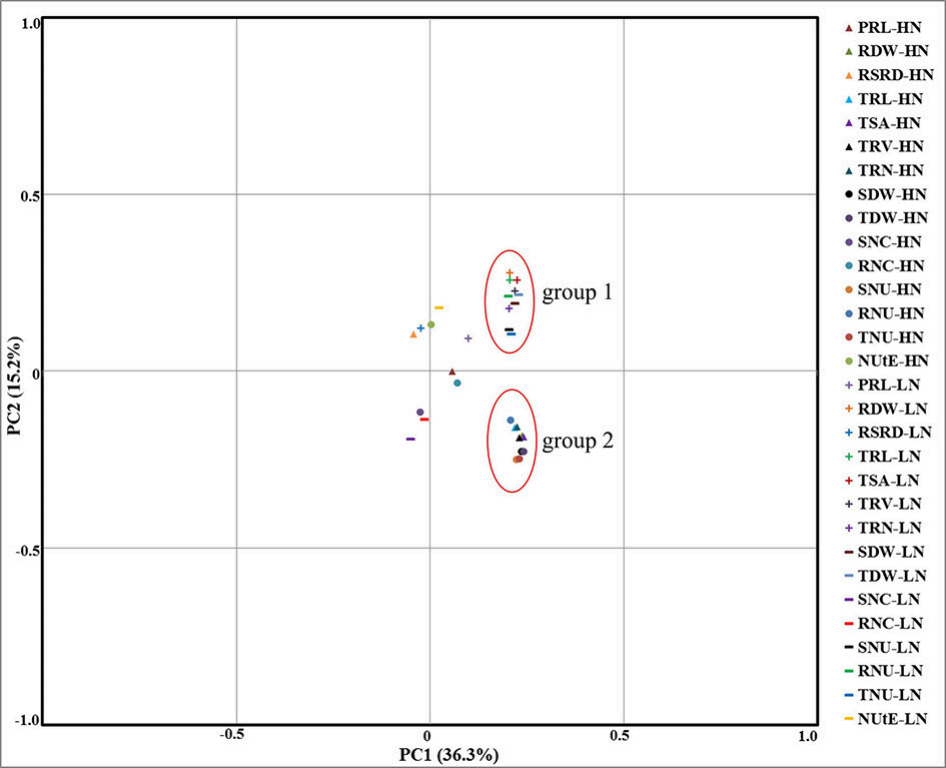
PCA of the RIL population for RM- and NUE-related traits evaluated in hydroponics under the conditions of HN (triangle and circle respectively) and LN (cross and horizontal line respectively) levels. Thirty traits were projected onto the first and second principal components. Two obvious groups indicated by red circles: group1 or group2 included all of the RM- and NUE-related traits (except PRL, RSRD, RNC, SNC, and NUtE) under LN or HN conditions, respectively. The traits had the greatest positive influence over the determination of PC1 (>0.2) were all located in the two groups. Group1 had positive influence over the determination of PC2 while group2 had negative influence. Traits in each group were all closely related and combined into two clusters distributed in two different N levels, respectively.

Principle component analysis (PCA) also showed that RM-related traits except PRL and RSRD were all closely related and combined into two groups distributed in two different N levels, respectively (Figure 1). In NUE-related traits, only DB and NU were both assigned into the two RM-related groups. Thus, all these results showed that RM-related traits (except for PRL and RSRD) were more likely to be genetically associated with DB and NU, but not with NUtE, indicating that RM-related traits may play an extremely important role in plant N uptake rather than in plant N utilization.

### Detection of QTLs associated with RM- and NUE-related traits

**Figure 2:**
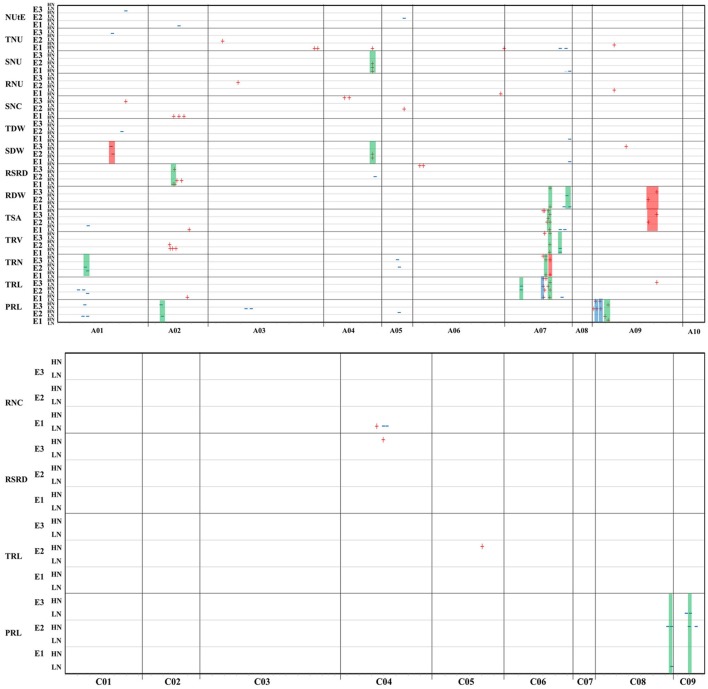
Genomic regions of 129 QTLs detected for RM-related traits and NUE-related traits across three independent hydroponics experiments (E1-E3) under HN and LN levels. For each QTL symbol, red sign (+) or blue sign (–), represents positive additive effect from ZS11 or No. 73290 alleles respectively and the horizontal ordinate indicate the peak position. The rectangles represent the sQTL: red for LN-specific sQTL, blue for HN-specific sQTL and green for constitutive sQTL.

Using the WinQTL cartographer software, a total of 129 identified QTLs (104 significant QTLs and 25 overlapping suggestive QTLs) associated with the investigated traits was detected on three environments, respectively. Among these, 91 QTLs were assumed to influence RM-related traits and 38 QTLs were assumed to influence NUE-related traits (Figure 2, Tables S2, S3). These QTLs were located across 12 of the 19 chromosomes in *B. napus* (including A01-A07, A09, C04, C05, C08, and C09). Of these, 45 (~35%) were located on the A07 linkage. More QTLs for NUE-related traits was detected under LN condition than that under HN level (7 under HN condition, 31 under LN condition), while similar number of QTLs for RM-related traits was detected under two N levels (43 under HN condition, 48 under LN condition; Table S3). Consequently, 56 out of 129 identified QTLs (~43% of the total number QTLs) were integrated into 23 sQTLs, which were repeatedly detected at least two environments or different N levels (Figure 2 and Table 4). Among these, 20 sQTLs were associated with RM-related traits and 3 sQTLs were associated with NUE-related traits. Notably, 16 out of 23 sQTLs were considered as “constitutive sQTL,” because they were detected under both HN and LN conditions. These results suggested that QTLs for RM-related traits are less affected than NUE-related traits on changeable environment under both HN and LN conditions.

**Table 4:** Summary of stable QTLs (sQTLs) for all investigated traits across all the environments.

**Category**	**Trait**	**QTL**	**Environment**		**Linkage group**	**Position (cM)**	**LOD**	**R^2^^a^ (%)**	**ADD^b^**	**Type^c^**
			**HN**	**LN**						
RM-related traits	PRL	sqPRL.A02	E1	E3	A02	21–34	2.3–2.7	4.9–5.4	−0.765 to −0.796	minor sQTL
		sqPRL.A09-1	E2,E3	–	A09	0.9–8.3	3.3–6.9	6.8–12.5	0.848 to 0.864	minor sQTL
		sqPRL.A09-2	E2,E3	–	A09	8.3–18.2	2.7–6.7	5.8–13.4	0.790 to 0.891	minor sQTL
		sqPRL.A09-3	E1	E1, E3	A09	18.3–32.8	2.8–4.0	5.2–7.8	0.746 to 0.926	minor sQTL
		sqPRL.C08	E2	E1	C08	115–124	3.7–4.8	7.2–9.9	−0.782 to −0.922	minor sQTL
		sqPRL.C09	E2	E3	C09	22.7–27.8	3.1–3.5	5.3–6.7	−0.576 to −0.846	minor sQTL
	TRL	sqTRL.A07-1	E2	E2	A07	24.7–28.6	2.2–3.7	4.5–7.7	−85.516 to –129.366	minor sQTL
		sqTRL.A07-2	E2,E3	–	A07	57.4–63	2.3–4.9	5.8–12.6	67.991 to 119.916	minor sQTL
		sqTRL.A07-3	E2	E1, E2, E3	A07	70.3–77	2.5–8.0	5.1–17.1	61.017 to 136.877	minor sQTL
	TSA	sqTSA.A07	E2,E3	E1,E2,E3	A07	69.7–76.2	2.4–8.0	5.0–17.1	4.130 to 12.658	major sQTL
		sqTSA.A09	–	E2, E3	A09	84.8–104.7	2.3–3.5	4.4–7.9	4.602 to 5.037	minor sQTL
	TRV	sqTRV.A07-1	E3	E1, E2	A07	71.3–75	3.1–4.6	7.3–9.1	0.029 to 0.047	minor sQTL
		sqTRV.A07-2	E1	E1	A07	86.7–98.8	2.3–3.1	4.8–6.3	−0.030 to −0.039	minor sQTL
	TRN	sqTRN.A01	E1	E2	A01	43.6–50.6	4.3–5.3	11.4–13.6	−65.052 to –114.203	major sQTL
		sqTRN.A07-1	E3	E1, E3	A07	65.5–70.3	4.6–5.9	9.4–11.6	38.030 to 54.769	major sQTL
		sqTRN.A07-2	–	E1, E3	A07	71.8–76.4	5.9	11.6–11.9	46.593 to 62.766	major sQTL
	RDW	sqRDW.A07-1	E3	E1	A07	73.7–79.2	3.0–3.8	6.7–7.8	0.002	minor sQTL
		sqRDW.A07-2	E2	E1	A07	101.3–111.2	2.8–4.4	6.4–9.2	−0.002 to −0.007	minor sQTL
		sqRDW.A09	–	E2, E3	A09	68.9–104.9	2.2–2.9	5.0–6.5	0.002	minor sQTL
	RSRD	sqRSRD.A02	E1	E1, E3	A02	36.2–48.2	2.2–6.8	4.3–14.8	0.003 to 0.010	minor sQTL
NUE-related traits	SDW	sqSDW.A01	–	E2, E3	A01	86.6–93	2.3–3.4	5.5–7.4	−0.008 to −0.013	minor sQTL
		sqSDW.A04	E1	E2	A04	78.5–83	2.9–3.5	6.4–7.5	0.009 to 0.020	minor sQTL
	SNU	sqSNU.A04	E1	E1, E2	A04	77.3–83	3.2–3.9	6.2–9.1	0.215 to 1.386	minor sQTL

a *Phenotype variation explanation of QTLs*.

b *Positive and negative values represented corresponding QTLs carried the favorable alleles from ZS11 and No. 73290, respectively*.

c *Represented the type of sQTL, minor sQTL or major sQTL*.

#### RM-related traits

A total of 70 significant QTLs and 21 overlapping suggestive QTLs, explained 4.3–17.1% of the phenotypic variance, were detected with LOD scores ranged from 2.2 to 8.0 for RM-related traits. 23 QTLs were detected for PRL, 57 for root size (RS: including TRL, TSA, TRV, TRN, and RDW) and 9 for RSRD (Tables S2, S3). These QTLs were integrated into 20 sQTLs, including 14 constitutive sQTLs, 3 HN-specific sQTLs and 3 LN-specific sQTLs. Six sQTLs were found for PRL that explained 4.9–13.4% phenotypic variation, including 2 HN-specific sQTLs detected on chromosome A09 (Table 4). 13 sQTLs were found from RS (3 for TRL, 2 for TSA, 2 for TRV, 3 for TRN and 3 for RDW), which included 4 major sQTLs (all sQTLs for TRN and one for TSA), 3 LN-specific sQTLs (for TSA, TRN and RDW, respectively) and one HN-specific sQTL for TRL. Besides, only one sQTL located on chromosome A02 was found for RSRD. Notably, most of these sQTLs (~69%) were densely distributed on 57.4–111.2 cM of chromosome A07, suggesting the presence of major genes regulating RM in this region. In particular, the LN-specific sQTLs for RDW (sqRDW.A09) was co-localized with the LN-specific sQTL for TSA (sqTSA.A09) on chromosome A09 (68.9–104.9 cM), though the two sQTLs had minor genetic effects explaining 4.4–7.9% of the phenotypic variations.

#### NUE-related traits

Relatively fewer identified QTLs were detected for NUE-related traits, including 34 significant QTLs and 4 overlapping suggestive QTLs, explained 5.5–12.6% of the phenotypic variance, with LOD scores ranged from 2.3 to 5.8. These QTLs included 6 QTLs for SDW, 2 for TDW, 10 for N concentration (NC, including RNC and SNC), 17 for N uptake (NU, including RNU, SNU, and TNU) and 3 for NUtE (Tables S2, S3). Of these QTLs, only three sQTLs were found, including one LN-specific sQTLs for SDW and two constitutive sQTLs (one each for SDW and SNU; Table 4). This LN-specific sQTL for SDW (sqSDW.A01) was located on 86.6–93 cM of chromosome A01 and explained 5.5–7.4% of phenotypic variation. The two constitutive sQTLs were co-localized on chromosome A04 (77.3–83 cM) and explained 6.2–9.1% of phenotypic variation.

### Detection of pleiotropic or specific QTL clusters for RM- and NUE-related traits revealed two NUE-improving approaches in rapeseed

**Figure 3:**
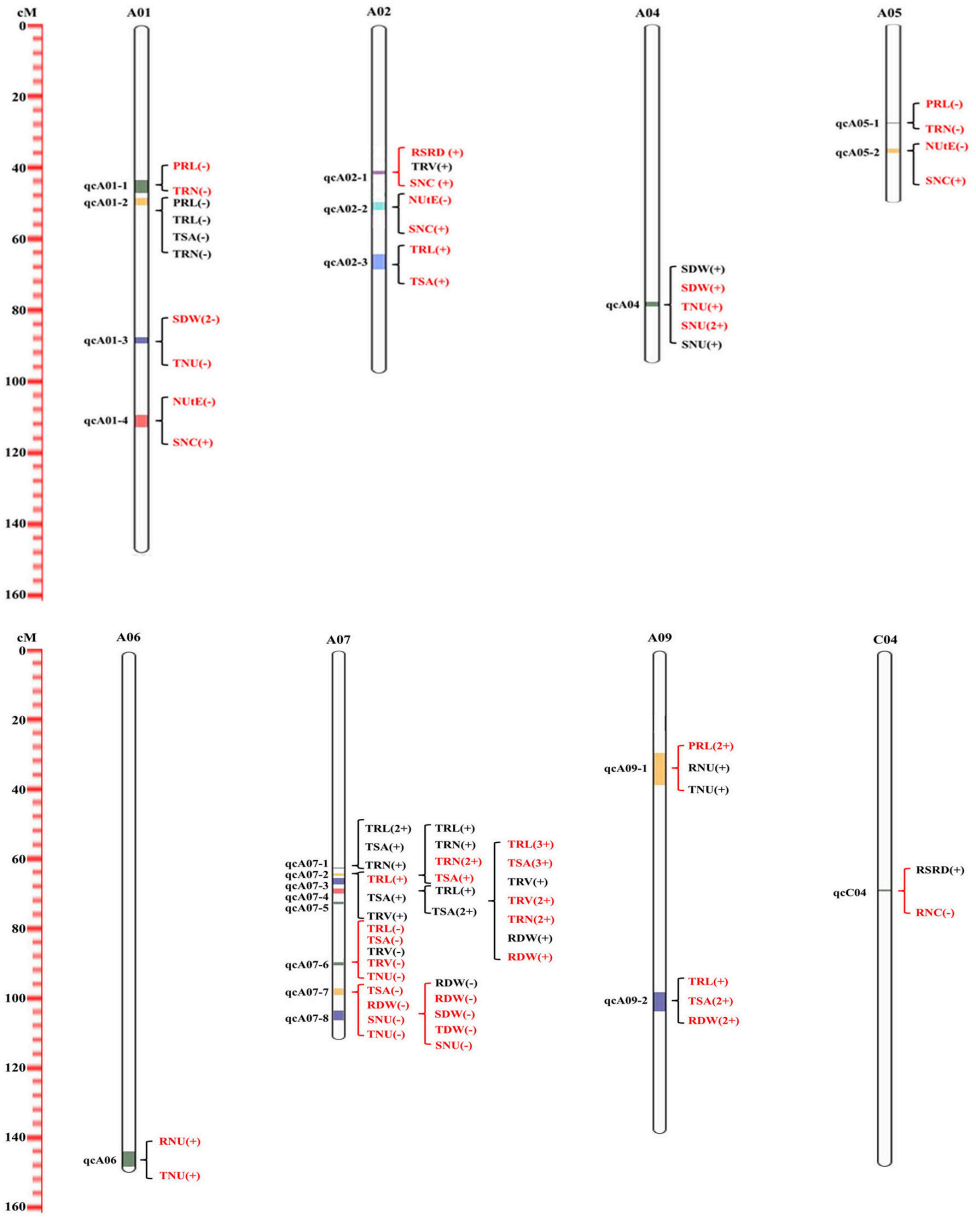
Identified QTL clusters for all investigated traits as revealed by meta-QTL analysis. The genetic distance where the QTL cluster located is represented by a vertical ruler and the chromosome number is marked on top. Each QTL cluster is indicated by the opening brace across all chromosomes and its name is give on the left of the chromosome. Red opening brace is marked if QTLs for QTLs for RM-related traits and NUE-related traits are located in a same cluster else black. The QTLs contained in a QTL cluster are given on the right of the chromosome and QTLs detected at HN levels are indicated in black, and at LN levels in red. The number between parentheses indicates how many experiments this QTL was detected in. The sign, (+) or (–), represents positive additive effects from ZS11 and No. 73290 alleles, respectively.

Most pairs of RM-and NUE-related traits showed significantly genetic correlation in Table 3, providing the potential to identify QTLs with pleiotropy or specificity for these traits. QTL meta-analysis was performed to find pleiotropic “QTL cluster.” As a result, a total of 83 identified QTLs (~64% of the total number) were integrated into 22 distinct pleiotropic QTL clusters (Figure 3, Table 5). The 22 distinct QTL clusters were distributed on eight chromosomes (A01, A02, A04-A07, A09, and C04) with a range of 2–13 identified QTLs. About 68% of these were distributed on chromosome A01 (four), A02 (three), and A07 (eight). Besides, QTL clusters detected for both RM-related traits and NUE-related traits were defined as “RM-NUE QTL cluster,” while QTL clusters that were exclusively detected for RM-related traits or NUE-related traits were named as “RM-specific QTL cluster” or “NUE-specific QTL cluster,” respectively. Thus, all of the 22 distinct QTL clusters were divided into 10 (~45%) RM-specific QTL clusters, 6 (~27%) NUE-specific QTL clusters, and 6 (~27%) RM-NUE QTL clusters.

**Table 5:** Information of the detected QTL clusters for all investigated traits in different environments.

**Category**	**Clouster**	**Treatment**	**Linkage group**	**Peak position (cM)**	**Confience interval (cM)**	**ADD^a^**	**QTL numbers included**	**sQTLs included**
RM-specific QTL cluster	qcA01-1	LN	A01	44.92	43.05–46.8	−	2	sqTRN.A01
	qcA01-2	HN	A01	49.34	48.27–50.41	−	4	sqTRN.A01
	qcA02-3	LN	A02	67.41	65.19–69.64	+	2	–
	qcA05-1	LN	A05	28.13	27.98–28.27	−	2	–
	qcA07-1	HN	A07	62.8	62.46–63.15	+	4	sqTRL.A07-2
	qcA07-2	HN/LN	A07	65.2	64.83–65.58	+	3	–
	qcA07-3	HN/LN	A07	67.2	66.23–68.18	+	5	sqTRN.A07-1
	qcA07-4	HN	A07	71.4	70.46–72.34	+	3	sqTRL.A07-3/sqTSA.A07/sqTRV.A07-1/sqTRN.A07-2
	qcA07-5	HN/LN	A07	73.91	73.6–74.22	+	13	sqTRL.A07-3/sqTSA.A07/sqTRV.A07-1/sqTRN.A07-2/sqRDW.A07-1
	qcA09-2	LN	A09	101.94	99.07–104.81	+	5	sqTSA.A09/sqRDW.A09
NUE-specific QTL cluster	qcA01-3	LN	A01	88.96	88.02–89.91	−	3	sqSDW.A01
	qcA01-4	LN	A01	112.01	110.16–113.85	±	2	–
	qcA02-2	LN	A02	51.31	50.12–52.49	±	2	–
	qcA04	HN/LN	A04	79.84	79.15–80.52	+	6	sqSDW.A04/sqSNU.A04
	qcA05-2	LN	A05	36.15	35.5–36.81	±	2	–
	qcA06	LN	A06	149.18	146.88–151.48	+	2	–
RM-NUE QTL cluster	qcA02-1	HN/LN	A02	41.71	41.15–42.27	+	3	sqRSRD.A02
	qcA07-6	HN/LN	A07	90.65	90.12–91.18	−	5	sqTRV.A07-2
	qcA07-7	LN	A07	99.05	98.02–100.09	−	4	–
	qcA07-8	HN/LN	A07	106.31	104.77–107.84	–	5	sqRDW.A07-2
	qcA09-1	HN/LN	A09	33.71	28.96–38.45	+	4	sqPRL.A09-3
	qcC04	HN/LN	C04	69.66	69.37–69.94	±	2	–

a *Positive and negative values represented corresponding QTLs carried the favorable alleles from ZS11 and No. 73290, respectively*.

#### Pleiotropic QTL clusters for RM-related traits

Among the ten RM-specific QTL clusters, half of them were located on chromosome A07 and the remainders were on A01 (two), A02 (one), A05 (one) and A09 (one) (Figure 3, Table 5). On chromosome A07, the five QTL clusters were densely distributed on 62.46–74.22 cM, comprising seven sQTLs (including three major sQTLs, one LN-specific sQTLs, one HN-specific sQTL as well as five constitutive sQTLs). Notably, the largest RM-specific QTL clusters, qcA07-5 included up to 13 identified RM-related QTLs and was co-localized with five stable QTLs (two major sQTLs, one LN-specific sQTL as well as four constitutive sQTLs), indicating that qcA07-5 have a higher possibility of contributing specifically to root development under both HN and LN levels. Besides, qcA09-2 was detected only under LN level and co-localized with two LN-specific sQTL (sqTSA.A09 and sqRDW.A09), suggesting that qcA09-2 may be closely related to root growth under LN stress.

#### Pleiotropic QTL clusters for NUE-related traits

For the six NUE-specific QTL clusters, five of them was detected only under LN level, indicating the presence of LN stress stimulated genetic mechanism for NUE (Figure 3, Table 5). Among them, the largest QTL cluster, qcA04, was co-localized with two stable QTLs (constitutive sQTLs for SDW and SNU, respectively), showing that qcA04 may specifically contribute to shoot development and N uptake. Besides, qcA01-3 was co-located with the LN-specific sQTL, sqSDWA01.

#### Pleiotropic QTL clusters for both RM- and NUE-related traits

Of the six RM-NUE QTL cluster, five clusters was detected under both HN and LN condition (Figure 3, Table 5). On the other hand, four RM-NUE QTL clusters were overlapped between RM and NU traits; two were between RM and DB and others were between RM and NC, but no were between RM and NUtE. These results indicated that the co-localization of RM-related traits and NUE-related traits was existed under both HN and LN conditions, while RM-related traits were closely related with plant N uptake instead of N utilization efficiency. Five RM-NUE QTL clusters (qcA02-1, qcA07-6, qcA07-7, qcA07-8, and qcA09-1) had same directions of additive-effects. Among them, three QTL clusters had the favorable alleles coming from No. 73290 (qcA07-6, qcA07-7, and qcA07-8), while two QTL clusters had the favorable alleles derived from ZS11 (qcA02-3 and qcA09-1). The three largest RM-NUE QTL clusters (qcA07-6, qcA07-7, and qcA07-8) were densely distributed on 90.12–107.84 cM of chromosome A07 and co-localized with two constitutive stable QTLs, sqTRV.A07-2 and sqRDW.A07-2, implicating the presence of major genes regulating root growth and plant N uptake in this region. In addition, qcA02-1 and qcA09-1 were co-localized with two constitutive stable QTLs, sqRSRD.A02 and sqPRL.A09-3, respectively.

Taken together, three types of QTL clusters (RM-NUE QTL clusters and RM or NUE-specific QTL clusters) implied two NUE improvement approaches were existed in rapeseed: RM-based and N utilization-based direct approaches. The credible genetic relationship between RM and NUE in rapeseed laid a solid theoretical foundation for RM-based approach to NUE genetic improvement. More importantly, more QTLs for RM-related traits (91) were identified than that for NUE-related traits (38) in this study, and all of the four major sQTLs and most of stable QTLs (20 out of 23) were detected to be related to RM traits under HN and/or LN levels, indicated that the manipulation of RM for improving NUE would be more feasible and reliable than regulating nitrogen efficiency directly in rapeseed.

## Discussion

### Genetic relationship between RM and NUE traits implied two NUE improvement approaches in rapeseed: RM-based AND N utilization-based direct approaches

Root system is the key place for N acquisition (Lynch, 1995). Illuminating the genetic relationship between RM and NUE traits could provide the basis of RM-based approach for NUE genetic improvement. Our study uncovered the tight connection between RM and NUE traits from two aspects. On the one hand, phenotypic correlation analysis and principal component analysis showed high genetic correlation between RM and NUE traits (Figure 1, Table 3). Generally, RM-related traits (except for PRL and RSRD) had high positive correlations with DB and NU irrespective of N levels, but no correlation or little correlation with NUtE under HN or LN level respectively. PCA also showed that RM-related traits (except for PRL and RSRD) and NUE-related traits (except for NC and NUtE) were assigned into the same group under HN and LN conditions, respectively (Figure 1, group 1 and group 2). These results indicated that RM, especially “root size,” play a critical role in plant N uptake, rather than in N utilization, which was consistent with the previous report on maize (Li et al., 2015). On the other hand, the co-localization of QTLs for RM- and NUE-related traits in this study further supports the tight connection between RM and NUE traits. Among the six RM-NUE QTL clusters, four overlapped between RM and NU, two between RM and DB and others between RM and NC, but no between RM and NUtE, and five of them were positive in which all the QTLs had same additive-effect directions (Figure 3, Table 5). These proved that N acquisition, rather than N utilization, is more likely correlated with root morphology.

In conclusion, the significantly phenotypic correlation and the co-localization of QTLs fully demonstrated the presence of credible genetic relationship between RM and NUE in rapeseed. However, the process of plant N utilization is complex, mainly including N uptake by roots and translocation, assimilation and remobilization inside the plant (Xu et al., 2012). RM and root uptake activity (including root vigor, root N transport, etc.) are the major determinants of N acquisition (Glass, 2003; Garnett et al., 2009; Xu et al., 2012), while the N translocation, assimilation and remobilization refer to N metabolism process (Xu et al., 2012). So, given the presence of six NUE-specific QTL clusters in this study (Figure 3, Table 5) and the credible genetic relationship between RM and NUE, our results provided evidences for two NUE improvement approaches in rapeseed: RM-based and N utilization-based direct approaches.

### Important pleiotropic QTL clusters used for NUE-improving in rapeseed

Both RM and NUE are complex traits, susceptible to growth environment, and difficult in directly measuring that restrict genetic improvement of NUE and RM in crops (Hochholdinger and Tuberosa, 2009). Evaluating these traits using the genomic approach and marker-assisted selection are worldwide challenges for NUE improvement (Coque et al., 2008; Liu et al., 2008; Li et al., 2015). In this study, 129 identified QTLs (including 23 stable QTLs and 4 major QTLs) were detected and 83 QTLs of them were integrated into 22 pleiotropic QTL clusters (Figures 2, 3, Tables 4, 5, Tables S2, S3). Five RM-NUE (qcA02-1, qcA07-6 to qcA07-8 and qcA09-1), ten RM-specific (qcA01-1, qcA01-2, qcA02-3, qcA07-1 to qcA07-5, and qcA09-2) and three NUE-specific (qcA01-3, qcA04, and qcA06) QTL clusters with same directions of additive-effect provided the potential to identify pleiotropic genes or different closely-linked genes controlling root development and N uptake. More importantly, the favorable alleles among these QTL clusters can be used directly for genetic improvement of NUE via a marker-assisted selection approach in rapeseed.

The three largest RM-NUE QTL clusters (qcA07-6, qcA07-7, and qcA07-8) were closely distributed on 90.12–107.84 cM of chromosome A07 and all had the favorable alleles coming from No. 73290. These QTL clusters co-localized with two constitutive stable QTLs, sqTRV.A07-2, and sqRDW.A07-2, suggesting that this region may play a key role in root growth and plant N uptake. Besides, three single-nucleotide polymorphisms (SNPs), significantly associated with RSRD, RDW and/or PRL, which were detected under low phosphorus (P) condition (Wang et al., 2017) were located in the similar genomic region with the three QTL clusters, respectively. In addition, Wang et al. (2017) identified another significantly associated SNP for root length, which was most likely co-localized with qcA02-1. Another QTL cluster, qcA09-1, located on 28.96–38.45 cM, was associated with PRL and RNU/TNU, whereas a QTL for PRL have previously been identified nearby (Zhang et al., 2016). Further, a significantly associated SNP for shoot concentration of Na discovered by Bus et al. (2014) was also localized in this genomic region. Consequently, we proposed that these positive RM-NUE QTL clusters were likely to be the common regions for regulating rapeseed RM traits across different genetic backgrounds, and further involved in plant N, P, and Na uptake.

Five RM-specific QTL clusters on chromosome A07 (from qcA07-1 to qcA07-5) were distributed on 62.46–74.22 cM. Seven sQTLs (including three major sQTLs) for RM-related traits were co-localized with these QTL clusters, suggesting the presence of major genes regulating RM in this region. Besides, under low P condition, Wang et al. (2017) identified six significantly associated SNPs with several root traits and Zhang et al. (2016) detected two QTLs for PRL and SDW that were located in the similar genomic region. Interestingly, all the QTLs distributed on 62.46–74.22 cM of chromosome A07 had the favorable alleles coming from the parental line ZS11, while on 90.12–107.84 cM from the parental line No. 73290, indicating that there are two distinct but important regulatory mechanisms for root development on chromosome A07 under this mapping population. On chromosome A01, qcA01-1 and qcA01-2 were distributed on 43.05–50.41 cM and the major stable QTL sqTRN.A01 was co-localized with these QTL clusters, suggesting the presence of major genes regulating RM in this region. The QTL uq.A1_1 for TRL detected by Zhang et al. (2016) using another genetic population was located in the similar genomic region. Thus, given the credible genetic relationship between RM and NUE, these important QTL clusters would have a considerable breeding value for RM-based NUE improvement in rapeseed, though no co-localization with NUE detected in this study.

### RM-based NUE improvement would be more effective than N utilization-based direct NUE improvement via marker-assisted selection in rapeseed

Our study suggested two different approaches for NUE improvement via marker-assisted selection in rapeseed: RM-based and N utilization-based direct approaches. Previous reports showed that RM-related traits investigated at an early development stage had significant links with NUE, yield and yield components (Li et al., 2015; Xie et al., 2017). These suggested that breeding varieties with large “root size” at seedling stage may be a promising approach to optimize N uptake efficiency in crops. RM-based NUE improvement provided a novel and exciting opportunity for the manipulation of RM via marker-assisted selection QTLs for improving NUE in rapeseed, though direct NUE improvement had made some achievements as the major approach for NUE breeding previously (Xu et al., 2012; Han et al., 2015). The credible genetic relationship between RM and NUE in this study laid a theoretical foundation for RM-based approach on NUE genetic improvement in rapeseed. Furthermore, in this study, ~2.4 times more QTLs were identified for RM-related traits (91) than that for NUE-related traits (38) and all of the four major QTLs and most of stable QTLs (20 out of 23) were detected to be related to RM traits under HN and/or LN levels, suggested that QTLs for RM-related traits are less affected than NUE-related traits on changeable environment under both HN and LN conditions. Thus, regulating RM to improve NUE via marker-assisted selection would be more feasible and reliable than regulating nitrogen efficiency directly in rapeseed. On the other hand, many co-localized QTLs associated with RM and NUE traits have been reported in crops (An et al., 2006; Coque et al., 2008; Liu et al., 2008; Li et al., 2015) and manipulation of RM via marker-assisted selection to improve nutrient efficiency has been successfully used in rice (Wissuwa, 2005; Pariasca-Tanaka et al., 2014; all for phosphorus use efficiency, PUE) and maize (Li et al., 2015, for NUE; Gu et al., 2016, for PUE). Moreover, Li et al. (2015) identified 53 advanced backcross-derived lines (ABLs) containing RM-NUE QTL clusters via marker-assisted selection of RM and the GY/NUE of these ABLs showed apparent mean increases of 13.8% under HN and of 15.9% under LN conditions, compared to recurrent background Wu312. All this indicated that manipulation of RM via marker-assisted selection to improve NUE would be more effective than marker-assisted selection for NUE directly in rapeseed.

Though nutrient absorption and root development of plants are different in hydroponic culture and field experiment (Chapman et al., 2012; Watt et al., 2013; Petrarulo et al., 2015), some of the experimental evidence available has shown their correlations (Lynch and Brown, 2001; Cui et al., 2008). Moreover, QTLs for root traits detected in hydroponics have been successfully used in nutrient efficiency improvement in field (Li et al., 2015, for NUE; Gu et al., 2016, for PUE). We deduced that the QTLs identified in this study could be used for marker-assisted selection in field, and corresponding examination will be conducted in the field in progress.

## Conclusions

Development of N efficient cultivars is beneficial for environment-friendly and sustainable rapeseed production. As shown in this study, RM had a significant positive phenotypic correlation with NUE and many RM-related QTLs were co-localized with QTLs for NUE, providing the credible genetic evidence for the significant associations between RM and NUE. In addition, 129 identified QTLs (including 23 stable QTLs and 4 major sQTLs) were detected and 83 QTLs of them were integrated into 22 pleiotropic QTL clusters. Five RM-NUE, ten RM-specific and three NUE-specific QTL clusters with same directions of additive-effect implied two distinct NUE-improving approaches (RM-based and N utilization-based directly) via marker-assisted selection in rapeseed. Importantly, all of four major QTLs and most of stable QTLs (20 out of 23) were detected to be related to RM traits under HN and/or LN levels, suggested that regulating RM to improve NUE would be more effective than regulating N efficiency directly. These pleiotropic QTL clusters also provided valuable genomic regions for RM-based or direct NUE-improvement. Whether the co-localization of QTLs was originated from pleiotropic genes or different closely-linked genes, fine mapping, RNA-seq and bioinformatics analysis should be conducted to confirm corresponding gene or genes in the future, which will lay a solid foundation for uncovering the genetic and molecular mechanism on NUE improvement in rapeseed.

## Author contributions

JW performed the data analysis and wrote the main manuscript text. XD designed and managed the experiments. JS contributed to the construction of molecular genetic map. XW and GL prepared the plant materials. HW reviewed the manuscript. All of the authors have read and approved the final manuscript.

### Conflict of interest statement

The authors declare that the research was conducted in the absence of any commercial or financial relationships that could be construed as a potential conflict of interest.

## Abbreviations

DB: dry biomass traits
HN: high-nitrogen
LN: low-nitrogen
NC: N concentration
NU: N uptake traits
NUE: nitrogen use efficiency
NUtE: N utilization efficiency
PRL: primary root length
SNP: single-nucleotide polymorphism
sQTL: stable QTL
RDW: root dry weight
RIL: recombinant inbred line
RM: root morphology
RNC: root N concentration
RNU: root N uptake
RSRD: root-shoot ratio in dry weight
SDW: shoot dry weight
SNC: shoot N concentration
SNU: shoot N uptake
TDW: total dry weight
TNU: total N uptake
TRL: total root length
TRN: total root number
TRV: total root volume
TSA: total root surface area.
